# Optimized Combination of Local Beams for Wireless Sensor Networks

**DOI:** 10.3390/s19030633

**Published:** 2019-02-02

**Authors:** Semyoung Oh, Young-Dam Kim, Daejin Park

**Affiliations:** 1 Department of Electronic and Communication Engineering, Air Force Academy, Cheongju 28187, Korea; tpaud12345@airforce.mil.kr; 2 Department of Electrical Engineering, Korea Advanced Institute of Science and Technology, Daejeon 34141, Korea; hohohahe@kaist.ac.kr; 3 School of Electronics Engineering, Kyungpook National University, Daegu 41566, Korea

**Keywords:** collaborative beamforming, analog uniform linear array, millimeter wave channel, simulated annealing

## Abstract

This paper proposes an optimization algorithm to determine the optimal coherent combination candidates of distributed local beams in a wireless sensor network. The beams are generated from analog uniform linear arrays of nodes and headed toward the random directions due to the irregular surface where the nodes are mounted. Our algorithm is based on one of the meta-heuristic schemes (i.e., the single-objective simulated annealing) and designed to solve the objective of minimizing the average interference-to-noise ratio (INR) under the millimeter wave channel, which leads to the reduction of sidelobes. The simulation results show that synthesizing the beams on the given system can form a deterministic mainlobe with considerable and unpredictable sidelobes in undesired directions, and the proposed algorithm can decrease the average INR (i.e., the average improvement of 12.2 dB and 3.1 dB are observed in the directions of $\frac{\pi }{6}$ and $\frac{2\pi }{3}$, respectively) significantly without the severe loss of signal-to-noise ratio (SNR) in the desired direction.

## 1. Introduction

Wireless sensor networks (WSNs) have been widely studied and applied for activity-monitoring applications in military [1,2], weather [3,4], and commercial [5,6] areas. To share monitored data among nodes in WSN, recent WSNs generally utilize the communication protocol designed for industrial, scientific, and medical (ISM) or lower frequency bands. However, as the demand of high-capacity and high-rate data exchange grows, the millimeter wave (mmWave) bands have also been considered as the promising spectrum resources [7,8,9,10]. Although these frequency bands suffer from the severe path loss caused by the atmospheric absorption and scattering [11], they are still attractive because their small wavelengths allow to use phased array architectures such as uniform linear arrays (ULAs) [12] and coprime arrays [13,14], which can be implemented in the WSN nodes [15,16,17].

Usually, the nodes are clustered within a small region and surrounded by far-away access points (APs) [18]. When one of them, (i.e., a sink node S), needs to establish a direct uplink with one of the APs, it is possible to solely use its own beam. However, because of the two reasons that the longer transmission may cause a more serious path loss [19,20], and the small physical size and low power supply of the nodes [21] limit the number of the radiating elements in ULAs, it is more efficient to combine multiple beams from the neighbor nodes by utilizing the concept of a collaborative or cooperative beamforming (CB). This approach was introduced in [22] to analyze the possibility of extracting the directive beam from uniformly distributed nodes, and the cases of Gaussian and arbitrary distributions are also analyzed in [23] and [24], respectively. In addition, Tsinos et al. proposed the novel methods to efficiently obtain the beamforming weight of the nodes [25,26,27], and Jayaprakasam et al. deeply surveyed the fundamentals and applications of the CB. These studies give us the verified result that the CB can provide a well-defined and deterministic mainlobe in the direction of a desired AP. Unfortunately, unpredictable sidelobes caused by the random positions of the nodes also affect unwanted results over the whole angular domain, which would result in the unacceptable levels of interference in the directions of undesired APs. To minimize these malignancies, some types of techniques have been suggested and examined in the various literatures. Ahmed et al. formulated the combinatorial optimization problem with the node-selection scheme to prune the sidelobes [28], Chen et al. utilized the decentralized cross-entropy optimization (CEO) having the significantly reduced complexity compared to [29], Sun et al. adjusted the excitation amplitude and phase of the nodes by the firefly algorithm [30], and Jayaprakasam et al. proposed the nondominated sorting genetic algorithm II (NSGA-II) to solve the multi-objective amplitude and phase optimization having the goal of minimizing the peak sidelobe level minimization and maximizing the directivity simultaneously [31]. Although these schemes showed the prominent effects on reducing the sidelobe levels in the undesired directions, they are confined to the case that the nodes are equipped with omni-directional antennas and operate in the conventional frequency bands, which provides the motivation of our paper.

**Figure 1:**
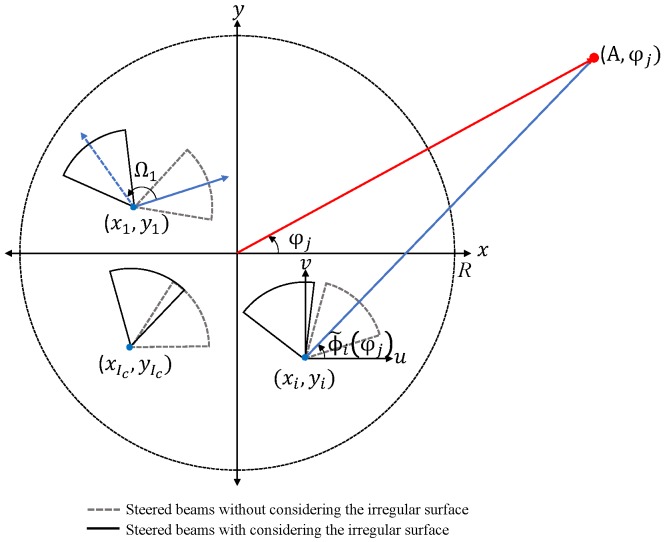
System model.

We assume the scenario that the nodes of the WSNs, operating in the mmWave band, are installed on the irregular surface of the practical sensing area and equipped with ULAs steering beams toward the desired direction, as shown in Figure 1. From simulation results, it is verified that the CB under the given scenario not only provides the power improvement in the desired direction, but also causes the considerable sidelobes. Thus, we design the combinatorial optimization for minimizing the average interference-to-noise ratio (INR). Unlike the node-selection and excitation-adjustment methods presented in [28,29,30,31], we utilize the beam-perturbation scheme which changes the steering angles of the beams by controlling phase shifters. Due to the strong directivity of the mmWave channel [11,12] and the intimate relation between the average INR and the sidelobe levels [28,29], it is expected that reducing the average INR, collected from the undesired APs, leads to the decrease of the sidelobe levels in the finite-discrete directions. To solve the problem in the efficient manner, we here introduce a meta-heuristic method such as the simulated annealing (SA) having the advantages of low memory capacity and scalability [32,33]. For the performance verification of our algorithm, we conduct numerical simulations in terms of the SNR, average INR, and complexity and show that the proposed algorithm remarkably reduces the average INR with the relatively low complexity. In summary, the main contribution of our paper is verifying that the CB is useful to increase the transmission range from the nodes being equipped with the ULAs to the desired AP and providing the optimization algorithm to lower the interference in the undesired APs under the mmWave channel.

## 2. System Model

We consider a WSN, operating in mmWave spectrum band, with a cluster having *I* sensor nodes $C=\left\{c_{1},\cdots ,c_{I}\right\}$ and $A\left(A\ll I\right)$ APs $A=\left\{a_{1},\cdots ,a_{A}\right\}$ which are in directions of $\varphi_{1},\cdots ,\varphi_{A}$, respectively. The sets of C and A can be used as the identifications (IDs) in the system. Under the assumption that the two terminals are coplanar on the x−y plane, the *i*th node is located at the coordinates of $\left(r_{i},\psi_{i}\right)=\left(\sqrt{x_{i}^{2}+y_{i}^{2}},tan^{-1}\left(\frac{y_{i}}{x_{i}}\right)\right)$, and the *j*th AP is a Euclidean distance $\rho_{i}\left(\varphi_{j}\right)=\sqrt{r_{i}^{2}+r_{A}^{2}-2r_{A}r_{i}cos\left(\varphi_{j}-\psi_{i}\right)}\approx r_{A}-r_{i}cos\left(\varphi_{j}-\psi_{i}\right)$ away from the corresponding node when $r_{i}\ll r_{A}$.

Due to the severe path loss within the cluster, the nodes need beamforming structures for the optimal directional link by considering operations of neighbor nodes. However, because they suffer from the limited battery-powered capacity, complex beamforming structures having multiple RF chains, (e.g., digital and hybrid schemes [34]), have disadvantages in terms of long-term operations of WSN. Thus, we apply a simple analog beamforming structure to each node, which is composed of a ULA with *N d*-wavelength spaced isotropic elements, *N* phase shifters, and a single RF chain. When the nodes are at the origin and the ULAs are parallel to the *x*-axis, their array factor can be simply defined as

(1) $$\mathrm{AF}\left(\phi \right)=\sum_{n=0}^{N-1}e^{jknd\left[\cos\;\phi -\cos\;\vartheta \right]}$$

where $\phi \in \left[-\pi ,\pi \right)$ is the azimuth angle, k=2π/λ is the propagation constant, λ is the wavelength at the operating frequency, and $\vartheta \in \left[-\pi ,\pi \right)$ is the steering angle of the ULA. However, in most cases, the positions of the nodes and the broadside directions of the ULAs are arbitrary. Therefore, we modify (1) to

(2) $${\mathrm{AF}}_{i}\left(\phi \;|\;\vartheta_{i}\right)=\sum_{n=0}^{N-1}e^{-jk\left\{\rho_{i}\left(\phi \right)-nd\left[\cos\left({\tilde{\phi }}_{i}\left(\phi \right)-\Omega_{i}\right)-\cos\;\vartheta_{i}\right]\right\}}$$

with the following assumptions: (1) the leftmost element of the ULA is placed on $\left(r_{i},\psi_{i}\right)$ following the random distribution; (2) $\left(r_{i},\psi_{i}\right)$ is the origin of the local u−v plane, where the *u* and *v* axes are parallel to *x* and *y* axes, respectively; (3) the included angle $\Omega_{i}$ between the *u*-axis and the line of the elements follows the uniform distribution of $U\left[-\pi ,\pi \right)$ due to the irregular surfrace where the nodes are mounted. In addition, ${\mathrm{AF}}_{i}\left(\cdot \right)$ is the array factor of the *i*th node; ${\tilde{\phi }}_{i}\left(\phi \right)=cos^{-1}\left({\hat{a}}_{i,j}\cdot \hat{u}\right)$ is the local azimuth angle with respect to the *u*-axis, which is simply converged to ϕ under the far-field condition; $\vartheta_{i}$ is the steering angle of *i*th node; ${\hat{a}}_{i,j}$ is the unit radial vector from $\left(r_{i}cos\psi_{i},r_{i}sin\psi_{i}\right)$ to $\left(r_{A}cos\phi ,r_{A}sin\phi \right)$; and $\hat{u}=\left[1\;0\right]$ is the unit vector in the direction of the *u*-axis.

To combine the beams coherently in the direction of $\phi =\varphi_{j}$, S starts sharing the data signal and the synchronization bits [35] with the $I_{c}$ collaborative nodes inside its maximum communication radius *R*, denoted as the $D=\left\{d_{1},\cdots ,d_{I_{c}}\right\}$. Afterward, without the prior knowledge of $\Omega_{i}$, each node $d_{i}$ steers the beam toward $\vartheta_{i}=\varphi_{j}$ and retransmits the signal mixed with the closed-loop phase offset $\Gamma_{i,j}=\rho_{i}\left(\varphi_{j}\right)$ [22], where the two beam-alignment status can be extracted from a reference position system such as the Global Positioning System (GPS) [36,37]. Then, without considering mutual coupling effects among the nodes, the combined array factor is given as

(3) $$\begin{array}{lll} {\mathrm{AF}}_{t}\left(\phi \right) & = & \sum_{i=1}^{I_{c}}{\mathrm{AF}}_{i}\left[\phi \;|\;\varphi_{j}\right]e^{jk\Gamma_{i,j}}\\ & = & \sum_{i=1}^{I_{c}}\sum_{n=0}^{N-1}e^{-jk\left\{\rho_{i}\left(\phi \right)-\rho_{i}\left(\varphi_{j}\right)-nd\left[\cos\left(\phi -\Omega_{i}\right)-\cos\varphi_{j}\right]\right\}}\\ & = & \sum_{i=1}^{I_{c}}\sum_{n=0}^{N-1}e^{jk\left\{2\sin\left(\frac{\varphi_{j}+\varphi }{2}-\psi_{i}\right)\sin\left(\frac{\varphi_{j}-\varphi }{2}\right)+nd\left[\cos\left(\phi -\Omega_{i}\right)-\cos\;\varphi_{j}\right]\right\}}, \end{array}$$

and the power pattern is also represented as

(4) $$P\left(\varphi \right)={\left|{\mathrm{AF}}_{t}\left(\varphi \right)\right|}^{2}.$$

**Figure 2:**
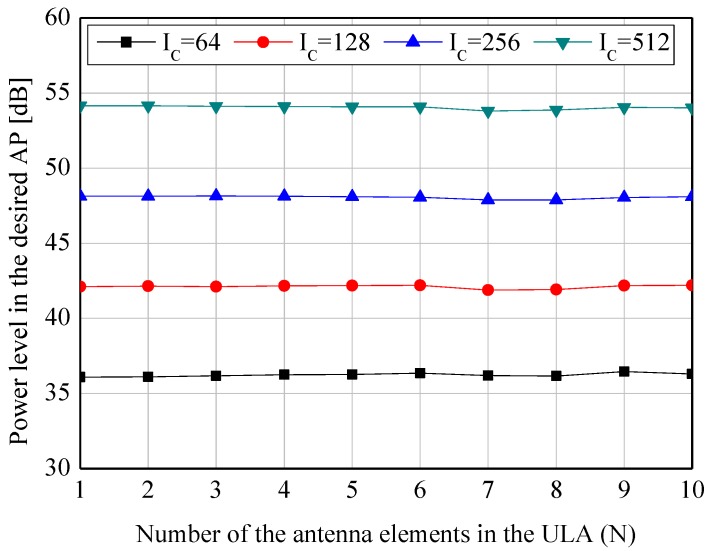
Simulation results of the system model with various *N* and $I_{C}$—Power levels, $P\left(\varphi_{1}\right)$, in the desired direction of $\varphi_{1}=0$.

**Figure 3:**
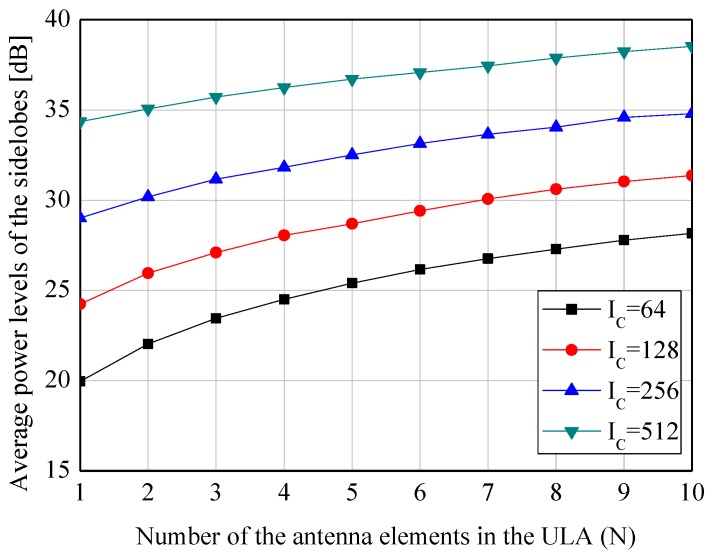
Simulation results of the system model with various *N* and $I_{C}$—Average power levels of the sidelobes, $\sum_{j=2}^{4}P\left(\varphi_{j}\right)/3$, over the undesired directions of $\varphi_{2}=\pi /3$, $\varphi_{3}=2\pi /3$, and $\varphi_{4}=\pi$.

Without the loss of generality, the directions of the desired and undesired APs are assumed to be $\varphi_{1}=0$, $\varphi_{2}=\pi /3$, $\varphi_{3}=2\pi /3$, and $\varphi_{4}=\pi$ henceforth. To verify the feasiblity of combining the multiple beams, the power levels observed in those directions are illustrated in Figure 2 and Figure 3. As seen in Figure 2, the higher value of $I_{c}$ (i.e., increasing the number of the nodes) significantly contributes to the improvement of $P\left(\varphi_{1}\right)$ due to the increased node density over the given area. However, it is also verified that *N* (i.e., the elements of the ULAs) is irrelevant to $P\left(\varphi_{1}\right)$. This phenomenon can be explained by the fact that the beams are randomly headed, and consequentially part of the beams cannot illuminate the desired AP. In addition, because of the previously mentioned reasons that the beams are randomly positioned and headed, the higher value of $I_{c}$ and *N* increases the average power level of the sidelobes in Figure 3.

## 3. Proposed Algorithm

As the first step of designing the practical algorithm to suppress the sidelobes, we consider the channel model first. Because the mmWave channel is sparse, it can be geometrically modeled as [38,39]

(5) $$\begin{array}{lll} h_{i,j}\left(\varphi_{j}\;|\;\vartheta_{i}\right) & = & \sqrt{\frac{1}{K_{i,j}^{\prime }}}\left[\sqrt{K_{i,j}}\beta_{i,j}^{1}{\mathrm{AF}}_{i}\left(\varphi_{j}\;|\;\vartheta_{i}\right)+\sum_{\ell =2}^{L_{i,j}}\frac{\beta_{i,j}^{\ell }}{\sqrt{L_{i,j}}}{\mathrm{AF}}_{i}\left(\varphi_{i,j}^{\ell }\;|\;\vartheta_{i}\right)\right]\\ & = & \sqrt{\frac{1}{K_{i,j}^{\prime }}}\left[\sqrt{K_{i,j}}\beta_{i,j}^{1}\sum_{n=0}^{N-1}e^{-jk\left\{\rho_{i}\left(\varphi_{j}\right)-nd\left[\cos\left(\varphi_{j}-\Omega_{i}\right)-\cos\;\vartheta_{i}\right]\right\}}\right.\\ & + & \left.\frac{1}{\sqrt{L_{i,j}}}\sum_{\ell =2}^{L_{i,j}}\sum_{n=0}^{N-1}\beta_{i,j}^{\ell }e^{-jk\left\{\rho_{i}\left(\varphi_{i,j}^{\ell }\right)-nd\left[\cos\left(\varphi_{i,j}^{\ell }-\Omega_{i}\right)-\cos\;\vartheta_{i}\right]\right\}}\right]. \end{array}$$

where $K_{i,j}$ is the Ricean K-factor between the node $d_{i}$ and the AP $a_{j}$, $K_{i,j}^{\prime }=1+K_{i,j}$, $\beta_{i,j}^{\ell }∼\mathrm{CN}\;\left(0,1\right)$ is the complex Gaussian channel coefficient of the *ℓ*th path, $\varphi_{i,j}^{\ell }∼U\left[-\pi ,\pi \right)$ is the angle of departure (AoD) of the the non-line-of-path (NLOS) paths, and $L_{i,j}∼U\left[2,4\right]$ is the integer number of the paths. Additionally, using (5), the combined signal at $a_{j}$ is given as

(6) $$y_{j}\left(\vartheta ,\Gamma_{j}\right)=\sum_{i=1}^{I_{c}}\sqrt{P_{i}}zh_{i,j}\left(\varphi_{j}\;|\;\vartheta_{i}\right)e^{jk\Gamma_{i,j}}+n,$$

where $\vartheta =\left[\vartheta_{1},\cdots ,\vartheta_{I_{c}}\right]$ and $\Gamma_{j}=\left[\Gamma_{1,j},\cdots ,\Gamma_{I_{c},j}\right]$ are the vectors of the steering angles and the phase offsets, individually; $P_{i}$ is the transmission power of the *i*th node; *z* is the data signal satisfied with $E\left\{{\left|z\right|}^{2}\right\}=1$; and $n∼\mathrm{CN}\left(0,\sigma_{n}^{2}\right)$ is the additive white Gaussian noise (AWGN) observed at $a_{j}$. Then, the INR for j≠1 are given as

(7) $$\begin{array}{ccc} \xi_{j}\left(\vartheta ,\Gamma_{1}\right) & = & \frac{{\left|y_{j}\left(\vartheta ,\Gamma_{1}\right)-n\right|}^{2}}{\sigma_{n}^{2}}\\ & = & \frac{{\left|\sum_{i=1}^{I_{c}}\sqrt{P_{i}}zh_{i,j}\left(\varphi_{j}\;|\;\vartheta_{i}\right)e^{jk\Gamma_{i,1}}\right|}^{2}}{\sigma_{n}^{2}}, \end{array}$$

and the average INR is defined as

(8) $$\begin{array}{lll} \Xi \left(\vartheta ,\Gamma_{1}\right) & = & \sum_{j=2}^{A}\xi_{j}\left(\vartheta ,\Gamma_{1}\right)/\left(A-1\right)\\ & = & \sum_{j=2}^{A}{\left|y_{j}\left(\vartheta ,\Gamma_{1}\right)-n\right|}^{2}/\left[\sigma_{n}^{2}\left(A-1\right)\right]\\ & = & \sum_{j=2}^{A}{\left|\sum_{i=1}^{I_{c}}\sqrt{P_{i}}zh_{i,j}\left(\varphi_{j}\;|\;\vartheta_{i}\right)e^{jk\Gamma_{i,1}}\right|}^{2}/\left[\sigma_{n}^{2}\left(A-1\right)\right]. \end{array}$$

To minimize the average INR, we open the combinatorial optimization problem with the objective function of $O\left(\vartheta_{k}\right)≜\Xi \left(\vartheta_{k},\Gamma_{1}\right)$ as follows:

(9) $$\vartheta_{opt}=\underset{\vartheta_{k}}{arg max}O\left(\vartheta_{k}\right).$$

Here, $\vartheta_{k}=\left[\vartheta_{1,k},\cdots ,\vartheta_{I_{c},k}\right]$ is the state vector to change the combination of the beams in a discrete manner, and $\vartheta_{i,k}\in \left\{-\pi +\frac{\pi }{N},\cdots ,-\pi +\frac{\pi \left(2m_{i,k}-1\right)}{N},\cdots ,-\pi +\frac{\pi \left(2N-1\right)}{N}\right\}$ is the steering angle having the approximate 3-dB beamwidth of 2π/N [40].

To find the global optimum of (9), all of the $N^{I_{c}}$ combinations should be exhaustively searched due to its non-convex characteristic. However, this approach imposes the impractical overhead on the system. Therefore, we utilize SA to ensure not getting stuck in local optima and approach good approximations to the global optimum efficiently. The SA emulates the metal annealing process, the goal of which is to reach a stable ground state. Similar to conventional methods (e.g., local search), the SA is basically based on the greedy transition from the current state $\vartheta_{k}$ to a better state $\vartheta_{k+1}$ for $\vartheta_{k}\neq \vartheta_{k+1}$, where both of the states are randomly created. However, even when $\Delta O=O\left(\vartheta_{k+1}\right)-O\left(\vartheta_{k}\right)\leq 0$ is not met, it uniquely allows the probabilistic transition toward a worse $\vartheta_{k+1}$ by the Metropolis criterion [41], such as

(10) $$exp\left(\frac{-\Delta O}{T_{f}}\right)\geq \eta ,$$

where $\eta ∼U\left[0,1\right]$, and $T_{f}$ is the current temperature. With the two transition mechanisms, the SA starts running the inner loop for the $R_{tot}$ iterations at the initial temperature $T_{0}$. After finishing the inner loop, the outer loop for cooling the temperature from $T_{0}$ to $T_{1}$ by the geometric temperature schedule of $T_{1}=T_{0}\times \rho$ with $\rho \in \left\{0.5,\cdots ,0.99\right\}$ is run, and another inner loop is repeated at $T_{1}$. This process continues until $T_{f}$ decreases to $T_{F}$, which is sufficiently low for the rare acceptance of new worse states. In addition, $R_{tot}$ at $T_{f}$ is adaptively determined as $R_{B}+\left\lfloor R_{B}F\right\rfloor$ to be close to the stationary distribution at the given temperature, where $R_{B}$ is the fixed number of the iterations, ⌊·⌋ is the flooring function, $F=1-exp\left[-\frac{\left(O_{H}-O_{L}\right)}{O_{H}}\right]$, and $O_{H}$ and $O_{L}$ are the highest and lowest values of the objective function at $T_{f-1}$, respectively [42]. Based on the above description, the SA can minimize the average INR effectively through the following steps.

**Step 1: Initialization.**

As the first step, S sets the control parameters of the SA, $T_{0}$, $T_{f}$, ρ, and μ, to the default values and broadcasts the *initialization message*. Here, μ is the predetermined acceptable INR in the undesired APs, and, for simplicity, it is equally set over the whole undesired APs. When each $d_{i}$ receives the message, it generates the beam the beam whose $\vartheta_{i,1}\in \vartheta_{1}$ is closest to $\varphi_{1}$. If $\varphi_{1}$ is in the middle between the two consecutive steering angles, one of them is selected. Go to **Step 2-2**. 

**Step 2: Optimization.**

**-**  **Step 2-1: Beam Perturbation(BP).**S starts the BP process from broadcasting the *beam perturb message* to D. When each $d_{i}$ receives this message, it steers the beam toward $\vartheta_{i,k}$ randomly chosen from $\left\{-\pi +\frac{\pi }{N},\cdots ,-\pi +\frac{\pi \left(2m_{i,k}-1\right)}{N},\cdots ,-\pi +\frac{\pi \left(2N-1\right)}{N}\right\}$. After switching the beam, the node responds by the *offer message* containing $d_{i}$ at a randomly delayed time [28], and S responds by the *approval message*. This step repeats until S receives the *offer message* from all elements of D or the scheduled time is over.
**-**  **Step 2-2: Sound.**D simultaneously transmit the *sounding messagez*. All of the APs measure and send their $\xi_{j}\left(\vartheta_{k},\Gamma_{1}\right)$ to a linked radio network controller (RNC). The RNC feeds $O\left(\vartheta_{k}\right)$ back to S, and S records $O\left(\vartheta_{k}\right)$.
**-**  **Step 2-3: Transition.** Once S receives the feedback signal, it checks whether the condition of $O\left(\vartheta_{k}\right)\leq \mu$ is met. If so, go to **Step 2-5**. Otherwise, the decision of the acceptance or rejection of the state transition is conducted. If $O\left(\vartheta_{k-1}\right)$ is not ready for this decision, go back to **Step 2-1**. If ready, $O\left(\vartheta_{k-1}\right)$ is replaced with $O\left(\vartheta_{k}\right)$ when the transition is accepted. After then, go back to **Step 2-1**.
**-**  **Step 2-4: Cooling Temperature.** The inner loop from **Step 2-1** to **Step 2-3** is repeated at the temperature $T_{f}$ for the *R* iterations. After the loop is terminated, $T_{f}$ is reduced to $\rho T_{f}$. Then, go back to **Step 2-1** and run another loop at $T_{f+1}$.
**-**  **Step 2-5: Optimization termination.** When the temperature finally reaches $T_{F}$ or $O\left(\vartheta_{k}\right)\leq \mu$ is met, the whole optimization process finishes, and the well-optimized combination of the beams $\vartheta_{opt}$ will be obtained.

## 4. Simulation Results

This section shows simulation results to evaluate the proposed scheme in terms of the SNR, average INR, and search complexity. Prior to the detailed studies, the system and channel are modeled as follows:

(1) System: Let the 64 sensor nodes be uniformly distributed [22] over a disk having the radius R=1λ and equipped with the $\frac{\lambda }{2}$ spaced ULAs. We assumed that they have the same transmission power $P=\frac{\sigma_{n}^{2}\eta }{I_{c}}$, where the power budget η is equal to 20 dB.
(2) Channel: Because the mmWave channels are commonly exposed to strong directivity [43], $\forall_{i,j}h_{i,j}\left(\cdot \right)$ are assumed to be under the line-Of-Sight (LOS) dominant scenario with $K_{i,j}=13.2$ dB.
(3) Optimization: In addition, in all simulations, $T_{0}$, $T_{f}$, $R_{B}$, and ρ are fixed to $10^{4}$, $10^{-5}$, $10^{2}$, and 0.8, respectively. As studied in [44,45], these parameters depend on the nature of the problem. Thus, a sensitivity analysis is used to choose the above values which are initial parameters to provide a good balance between exploration and exploitation of the search space. We omit the detailed procedure here due to the limited pages of this paper. Unfortunately, this empirical approach may result in the considerable simulation time consumption. Thus, an analytical and systematic method to determine the control parameters will be researched in our next paper.

**Figure 4:**
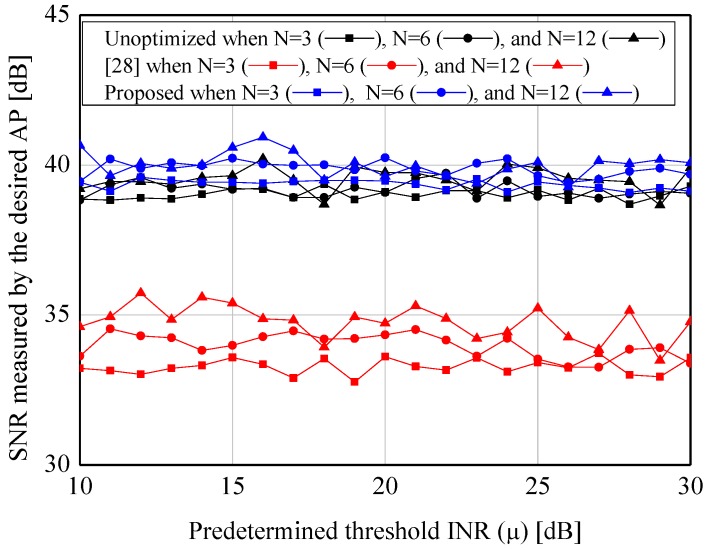
Performance of the sidelobe reduction algorithm—$\xi_{1}\left(\vartheta_{opt},\Gamma_{1}\right)$.

**Figure 5:**
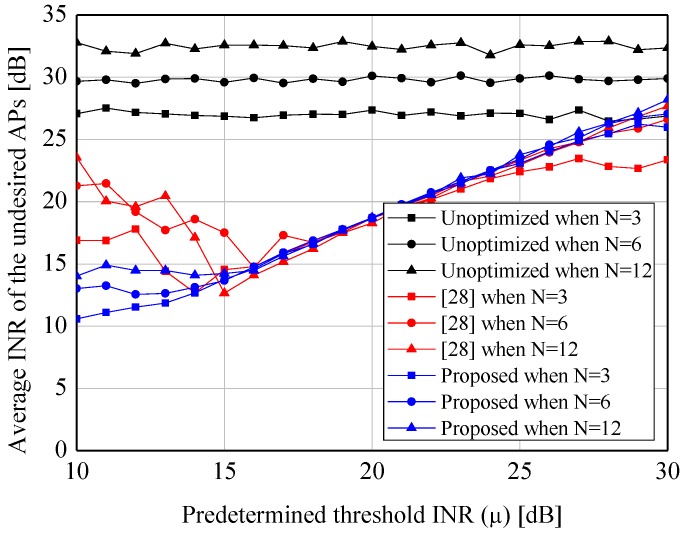
Performance of the sidelobe reduction algorithm—$\Xi \left(\vartheta_{opt},\Gamma_{1}\right)$.

**Figure 6:**
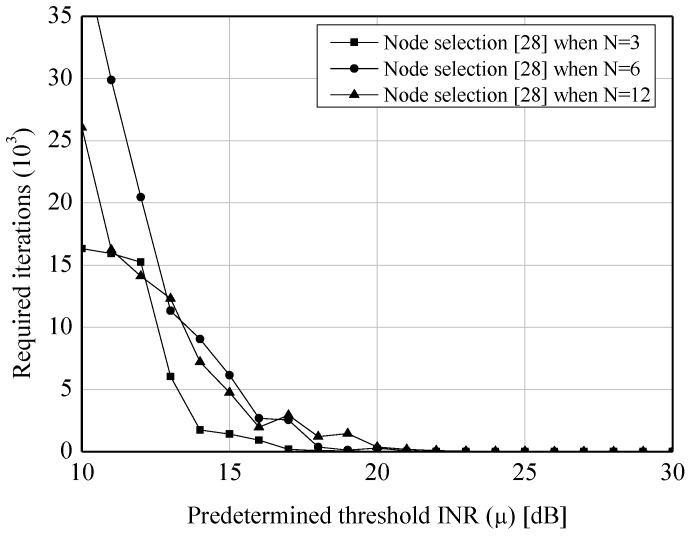
Performance of the sidelobe reduction algorithm—Search complexity for the node selection scheme [28].

**Figure 7:**
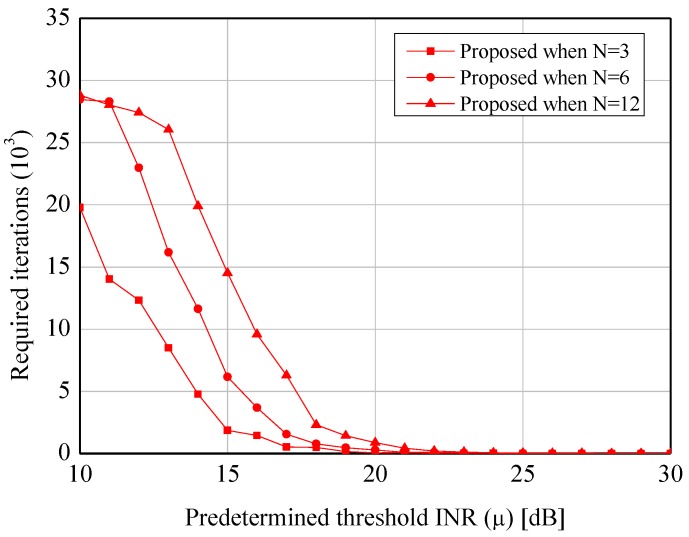
Performance of the sidelobe reduction algorithm—Search complexity for the proposed scheme.

Figure 4 and Figure 5 show the average INRs and SNRs to verify the validation of the proposed algorithm. From the two figures, the appreciable reductions of the average INRs (e.g., 15.8 dB when N=3 and μ=10 dB) are observed when our algorithm is applied. Especially, compared to the node selection scheme in [28], which is widely used to suppress the sidelobes in the CB, the proposed algorithm provides the better performance under the lower predetermined threshold (i.e., μ≤15 dB) in virtue of its probabilistic greedy exploration. Furthermore, while the former causes the severe loss of the SNRs due to its innate characteristic of node selection, the latter provides the constant SNR regardless of *N* and μ. In addition, Figure 6 and Figure 7 show the complexity of [28] and the proposed scheme. As expected, both of the approaches require the smaller number of iterations when the higher μ is set. However, as μ is decreased, the proposed algorithm requires more iterations than [28]. It can be explained by the fact that the core of our algorithm (i.e., SA) would spend many iterations to approach μ as much as possible.

## 5. Conclusions

In this paper, we investigated the combination of the randomly scattered and headed local beams for overcoming the restrictions such as the severe path loss of the mmWave channel and low power storages due to the limited system size of remotely installed sensor nodes and newly designed the optimization algorithm to effectively decrease the average INR over the undesired APs, resulting in the reduction of the sidelobes of the synthesized beam. The simulation results showed that the proposed algorithm can be considered as an effective way to establish the long-distance transmission with the low interference.

## Abbreviations

The following abbreviations are used in this manuscript:

ECG	Electrocardiogram
MLII	Modified Lead II
